# The 19 Fr. Intrauterine Bigatti Shaver (IBS®): a clinical and technical update

**Published:** 2018-09

**Authors:** G Bigatti, SH Ansari, W Di

**Affiliations:** Sino European Life Expert Centre, Shanghai Jiaotong University Affiliated Renji Hospital.160 Pujian Rd. Shanghai, China; Department of Obstetrics and Gynecology, Day General Hospital, Tehran Iran; Chief Director of OBGYN Department of Shanghai Jaotong University Affiliated Renji Hospital,160 Pujian Rd. Shanghai, China.

**Keywords:** IBS® Intrauterine Bigatti Shaver, operative hysteroscopy, new instrumentation, shaver technique

## Abstract

In this case report we describe the first two surgeries conducted with the 19 Fr. IBS®. The indication for operative hysteroscopy in both patients was the removal of polyps. The size of the polyps was between 15 mm and 20 mm with a mean resection time of 40 seconds. Normal saline solution (500 ml) was used with a negative fluid loss (100 ml). Both operations were successfully performed under general anaesthesia and no speculum, no tenaculum and no dilatation of the cervical canal were necessary.

## Introduction

Since the first operative hysteroscopy with the Shaver technique was performed, questions over conventional resectoscopy as the gold standard for major hysteroscopic operations arise (Di Spezio Sardo et al., 2008). In this report, we detail 2 operations performed with the IBS® and discuss the suitability of using a 19 Fr. IBS® in an office setting.

## Case report

### Ethical permit

The institutional ethical committee approved the research and all patients gave their informed consent. The indication for the two operative procedures was polypectomy.

### Patient inclusion and procedure

Both cases were performed under general anaesthesia, but no dilatation of the cervical canal was needed. In the second case we only dilated the inner cervical ostium using a 4 mm forceps introduced into the operative strait channel of the 19 Fr. Optics and then we entered the uterine cavity. A standard gynaecological set-up was used in the operating room.

**Figure 1:**
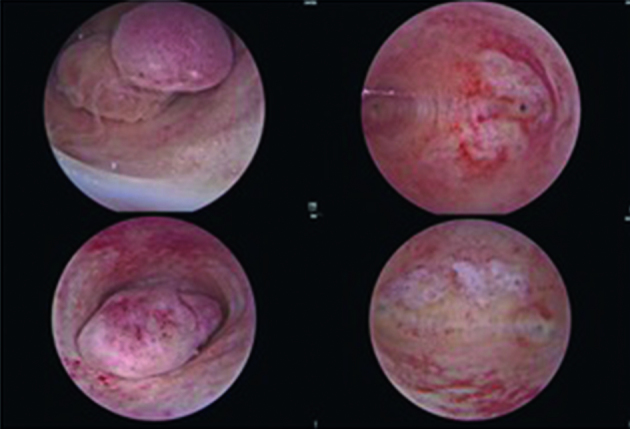
9Fr. IBS® before and after polypectomy.

Fluid balance, total operating time and resection time were recorded during the intervention. The size of the polyps was between 15 mm and 20 mm with a mean time of resection of 40 seconds. 500 ml of normal saline solution was used with a negative fluid loss of 100 ml (Table I). Both procedures were performed using the 19 Fr. IBS® and no complications occurred (Fig. 1).

**Table I:** 19 Fr. IBS® Patients details

Age	Spontaneous Deliveries	Diagnosis	Operation Time	Resection time	Fluid Used	Fluid deficit
54	3	20 mm Polyp	16 min	30sec	500 ml	100 ml
47	1	15 mm Polyps	8 min	50sec	500 ml	100 ml

All patients underwent general or regional anaesthesia, and a standard gynaecological set up was used in the operating room.

The first patient was a 53 - year - old lady with a history of 3 vaginal deliveries. A 2 cm polyp was found at ultrasound and was confirmed by diagnostic hysteroscopy.

The second patient was a 47 - year - old lady with a history of 1 vaginal delivery. She was suffering of menorrhagia and two 15 mm polyps were diagnosed by ultrasound and confirmed by diagnostic hysteroscopy. All the operations, except two, were performed using the standard 24 Fr. Optics as described in the original technique (Bigatti, 2011; 2018).

### Instruments and detailed intervention

Originally the Shaver technique was studied in order to approach large size pathology under general anaesthesia.

**Figure 2:**
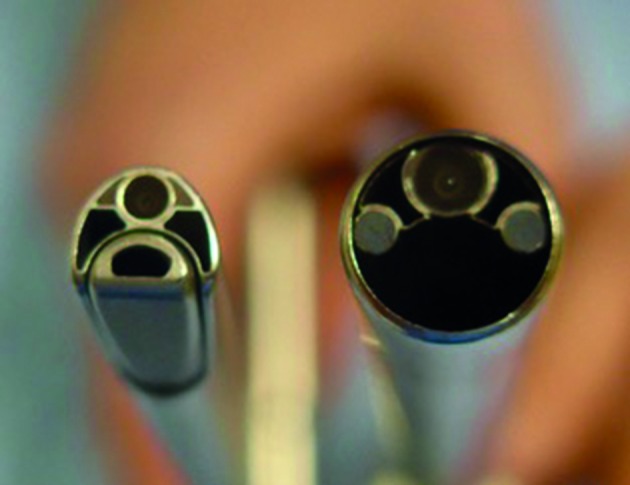
19Fr. IBS® Optics -24 Fr. IBS® Optics (KARL STORZ SE & Co. KG)

One of the limitations of the 24 Fr. optics has been its large size (Fig. 2). Notwithstanding a reduction in size compared to the 26 Fr. resectoscope, the IBS® large size discouraged its use in an office set up.

**Figure 3:**
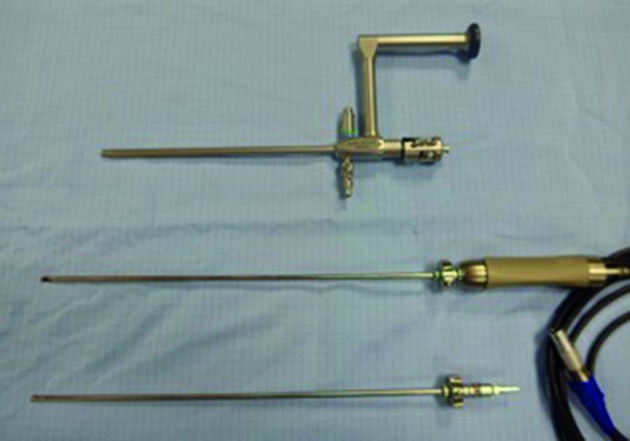
19Fr. IBS®: 90° angulated 6°Optics (KARL STORZ SE & Co. KG) single flow with strait operative channel with Obturator in place - Rigid shaving system - Blade

The new 19 Fr. IBS® featured a 90° - angulated 6° optics (Karl Storz SE & Co.KG) single flow with an additional strait operative channel (Fig. 3). Along this channel a special obturator with a round ergonometric tip was introduced in order to facilitate, during the vaginoscopy approach, the optical entrance into the cervical canal and into the uterine cavity. Once established in the desired position, the obturator was removed and replaced by the rigid shaving system. The rigid shaving system did not change in its design and consisted in two hollow reusable metal tubes fitting to each other The inner tube rotated within the outer tube and was connected to a handheld (Drill cut-X® Karl Storz SE & Co.KG) motor drive unit (Unidrive® S III by Karl Storz SE & Co.KG) and to a roller pump (Hysteromat E.A.S.I.® Karl Storz SE & Co.KG) controlled by a foot pedal.

The intrauterine pressure and the outflow were automatically checked and maintained by the Hysteromat E.A.S.I.® pump. The average of 2100 rotation power per minute was used. No speculum, no tenaculum and no dilatation of the cervical canal were necessary. At vaginoscopy the panoramic optic with inflow channels connected to the Hysteromat E.A.S.I.® pump was inserted into the uterine cavity. For irrigation conventional physiologic saline solution was used. The settings on the Hysteromat E.A.S.I.® pump display have been: intrauterine pressure of 90 mm/Hg, aspiration of 220/230 ml per minute. After the pathological site was visualized the obturator was removed and the rigid shaving system connected to the motor drive unit and to the Hysteromat E.A.S.I.® pump was introduced into the operative channel. Aspiration started only when the pedal of the roller pump was pressed, preventing the collapse of the uterine cavity due to the massive outflow. The rotating and oscillating movement of the inner blade of the shaving system cut the tissue allowing its aspiration. The specimens were collected for histology into plastic container provided with a filter normally used for dilatation and curettage. Thanks to this new instrument and revolutionary technique almost 90% of all major hysteroscopic procedures can be performed in a single step.

## Discussion

The Intrauterine Bigatti Shaver IBS® was developed with the intention to improve the results of conventional resectoscopy reducing by the number of complications (Witz et al., 1993; Schäfer et al., 2005; Van Kruchten et al., 2010; Pasini et al., 2001; Jansen et al., 2000). With the use of the IBS® we were able to remove the tissue chips at the same time as resection. With this, operative Hysteroscopy, always performed under visual control, esulted to be much faster and with a low complication rate. In the first case we described, we reported only one event (0.1%) of uterine perforation, 5 cases (0.7%) of intrauterine bleeding requiring bipolar coagulation and 5 cases (0.7%) of fluid overload treated with a diuretic therapy. No injuries to pelvic or abdominal organs or postoperative infections occurred. In addition, the IBS® proved to have a very fast learning curve compared to conventional bipolar resectoscopy (Bigatti et al., 2014). Another problem of the bipolar technique was that more than half of the uterine perforations were entry related due to the large diameter of conventional resectoscopes. With the 24 Fr. Optics we reported an improvement in the entry related complication rate with an incidence of 0.9% (Bigatti et al., 2012). In order to reduce this kind of complications and to promote the utilization of the Shaver in an office set-up we have implemented our technique with a new 19 Fr. Optical system. As previously mentioned, both cases were performed under general anaesthesia, but no dilatation of the cervical canal was necessary. We chose to operate under general anaesthesia because it was the first time that the 19 Fr. Optics was being used. Considering the short time for the whole procedure and the fact that we entered the uterine cavity by vaginoscopy without any need of dilatation in-office use, of the Shaver technique should be considered. In an office setting the use of an IBS® demonstrated its efficacy allowing for a to be a fast, clean, safe and precise procedure. Importantly, we started to remove the polyp from top to base of the pedicle without damaging the surrounding endometrium and no bleeding was observed. Furthermore, a randomized multicentre control study confirmed that a reduction of the instrument diameter improves the accessibility of office diagnostic hysteroscopy (Campo et al., 2005). In addition, by a statistical analysis of 530 diagnostic office mini-hysteroscopies in infertile patients proved that using an atraumatic insertion technique, watery distension medium and a new generation of mini-hysteroscopic endoscopes, hysteroscopy can be performed in an office set-up without any form of anaesthesia and with a high patient satisfaction (Campo et al., 1999). With regard to the evaluation of histopathological specimens, we found that morcellation did not affect the diagnosis as it has been previously reported (Franchini et al., 2015).

## Conclusion

In pre-selected patients with the right indication, the Shaver technique with the 19 Fr. Optics could be performed in an office environment easing the performance of the procedure to a single step intervention. Even if the number of cases is limited the paramount goal of this paper is to present and describe this innovative technique in order to promote its use and advantages among hysteroscopists worldwide.

Further studies with larger number of patients are expected in order to evaluate the effectiveness of the 19 Fr. Shaver office approach.

## Declaration of interest:

The authors report no declarations of interest and confirm that they have obtained the written permission of the patient whose case is being presented.

## References

[1] Bigatti G (2011). IBS® Integrated Bigatti Shaver, an alternative approach to operative hysteroscopy. Gynecol Surg.

[2] Bigatti G, Ferrario C, Rosales M (2012). IBS® Integrated Bigatti Shaver versus conventional bipolar resectoscope: a randomised comparative study.. Gynecol Surg.

[3] Bigatti G, Franchetti S, Rosales M (2014). Hysteroscopic Myomectomy with the IBS® Integrated Bigatti Shaver versus conventional bipolar resectoscope: a retrospective comparative study.. Gynecol Surg.

[4] Bigatti G The Shaver Technique for Operative Hysteroscopy In: Tinelli A, Alonso Pacheco L, Haimovich S (eds.) Hysteroscopy. Springer, Cham 2018; Chapter 55: 635-648

[5] Campo R, Van Belle Y, Rombauts L (1999). Office mini-hysteroscopy.. Hum Reprod Update.

[6] Campo R, Molinas CR, Rombauts L (2005). Prospective multicentre randomized controlled trial to evaluate factors influencing the success rate of office diagnostic hysteroscopy.. Hum Reprod.

[7] Di Spezio Sardo A, Mazzon I, Bramante S (2008). Hysteroscopic myomectomy: a comprehensive review of surgical techniques.. Hum Reprod Update.

[8] Franchini M, Zolfanelli F, Gallorini M (2015). Hysteroscopic polypectomy in an office setting: specimens quality assessment for histopathological evaluation.. Eur J Obstet Gynecol Reprod Biol.

[9] Jansen FW, Vredevoogd CB, van Ulzen K (2000). Complication of hysteroscopy: a prospective, multicentre study.. Obstet Gynecol.

[10] Pasini A, Belloni C (2001). Intraoperative complications of 697 consecutive operative hysteroscopies.. Minerva Ginecol.

[11] Schäfer M, Von Ungern-Sternberg BS, Wight E (2005). Isotonic fluid absorption during Hysteroscopy resulting in severe Hyperchloremic Acidosis.. Anaesthesiol.

[12] Van Kruchten PM, Vermelis JM, Herold I (2010). Hypotonic and isotonic fluid overload as a complication of hysteroscopic procedures: two case reports.. Minerva Anestesiol.

[13] Witz CA, Silverberg KM, Burns WN (1993). Complications associated with absorption of hysteroscopic fluid media.. Fertil Steril.

